# Estimation of the HIV Basic Reproduction Number in Rural South West Uganda: 1991–2008

**DOI:** 10.1371/journal.pone.0083778

**Published:** 2014-01-03

**Authors:** Rebecca N. Nsubuga, Richard G. White, Billy N. Mayanja, Leigh Anne Shafer

**Affiliations:** 1 Medical Research Council/Uganda Virus Research Institute, Uganda Research Unit on AIDS, Entebbe, Uganda; 2 Department of Infectious Disease Epidemiology, London School of Hygiene and Tropical Medicine, London, United Kingdom; 3 Health Sciences Center, University of Manitoba, Winnipeg, Manitoba, Canada; University of Buea, Cameroon

## Abstract

**Background:**

The basic reproduction number, 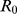, is one of the many measures of the epidemic potential of an infection in a population. We estimate HIV 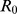 over 18 years in a rural population in Uganda, examine method-specific differences in estimated 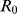, and estimate behavioural changes that would reduce 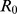 below one.

**Methods:**

Data on HIV natural history and infectiousness were collated from literature. Data on new sexual partner count were available from a rural clinical cohort in Uganda over 1991–2008. 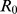 was estimated using six methods. Behavioural changes required to reduce 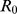 below one were calculated.

**Results:**

Reported number of new partners per year was 0 to 16 (women) and 0 to 80 (men). When proportionate sexual mixing was assumed, the different methods yielded comparable 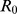 estimates. Assuming totally assortative mixing led to increased 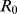 estimates in the high sexual activity class while all estimates in the low-activity class were below one. Using the “effective” partner change rate introduced by Anderson and colleagues resulted in 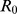 estimates all above one except in the lowest sexual activity class. 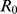 could be reduced below one if: (a) medium risk individuals reduce their partner acquisition rate by 70% and higher risk individuals reduce their partner acquisition rate by 93%, or (b) higher risk individuals reduce the partner acquisition rate by 95%.

**Conclusions:**

The estimated 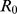 depended strongly on the method used. Ignoring variation in sexual activity leads to an underestimation of 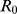. Relying on behaviour change alone to eradicate HIV may require unrealistically large reductions in risk behaviour, even though for a small proportion of the population. To control HIV, complementary prevention strategies such as male circumcision and HIV treatment services need rapid scale up.

## Introduction

The basic reproduction number, 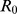, of an infection, is the mean number of secondary cases a single infected case will cause in a fully susceptible population. In disease modelling, 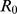 helps determine whether or not an infectious disease will spread through a population. When 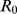 is below one, the infection will die out. If 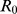 is greater than one, the infection may spread in a population. In general, the larger the value of 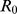, the harder it is to control the infection.

Various methods have been proposed to estimate 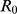 in HIV epidemics. These include: a method calculating 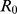 as a product of transmission probability, partner change rate and duration of infectiousness [1]–[3], a method assuming different transmission probabilities by HIV stage and by gender [4], a method with HIV stage progression probabilities [5], methods with different sexual activity classes [6], [7], and combinations of some of the above.

For populations which are no longer completely susceptible, the effective reproductive number, 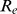, can be used to estimate the average number of secondary infections resulting from each case. We estimated 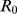 using a number of different methods to give an idea of the possible range of values of estimated 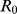 and hence show how difficult it may be to eradicate HIV. If desired, 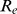 could be estimated from 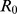 and a combination of other factors such as the proportion susceptible in a given period.

The aim of this work was to use different methods to estimate the hypothetical 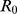, which would give the maximum value for R_e_, in an HIV epidemic over 18 years in a rural population in South West Uganda; examine the reduction in partner change rates required to lower 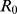 below one and hence potentially eradicate HIV; and to assess the effect of various scenarios of reductions in partner change rate on 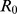.

## Methods

### Ethics

This work is secondary analysis of data obtained from a rural clinical cohort (RCC). The RCC study was approved by the Uganda Virus Research Institute Institutional Review Board and the Uganda National Council for Science and Technology.

Most estimation methods of 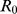 of HIV require data on the duration of infectivity of infected individuals, the infectiousness of the virus and new partner formation rates. Partner turnover changes over time, hence 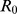 will be estimated for each survey year. We considered a range of methods which use the above parameters and are commonly used in literature for calculating R_0_.

### Data

The Medical Research Council (MRC)/Uganda Virus Research Institute (UVRI) research unit on AIDS has followed a “General Population Cohort” (GPC) in South West Uganda since 1989 [8] and in 1990 established a “Natural History Cohort” (NHC), later known as a “Rural Clinical Cohort” (RCC). Initially, the GPC comprised 15 villages; 10 were added in 2000. The RCC, a subset of the GPC, was setup to more closely follow HIV positive cases and negative controls. The cohort was described in [9], but in brief; initially a random selection of a third of prevalent HIV-positive and all incident HIV-positive adults identified from the GPC were invited to join the cohort. HIV-negative controls matched for age, sex and village of residence were randomly selected from the GPC and also invited to join the cohort. Later on with the introduction of ART, other HIV-positive individuals identified at the study clinic and met the inclusion criteria were also invited to join the cohort[10]. In total 192 HIV-positives and 222 HIV-negatives were enrolled before 2000; while 239 HIV-positives and 56 HIV-negatives were enrolled from 2000 to 2008.

HIV Transmission probabilities and duration of infectiousness were obtained from literature [11] (Table S1), as well as from the cohort data [12]–[14]. Background mortality, used in one of the methods to help estimate duration of infectiousness, was estimated from the GPC using the Brass relational life table system [15] (Details, Appendix S1).

### Partner change rate

Partner change rate was estimated from the RCC where the number of new sexual partners were collected each quarter. The annual partner change rate was calculated by summing the new partners per quarter over four quarters. In cases of missing quarterly data for an individual, annual partner change rate was calculated as:

4 x (summed new partners over the non-missing quarterly reports)/(number of quarters in the year with non-missing reports). The lowest sexual activity risk category coded “low” included those reporting zero or one new partner in a year. The medium category included those reporting more than one to two new partners in a year, and the highest risk category included those reporting more than two new partners. Since the RCC is not representative of the population, to estimate the population level sexual behaviour from the RCC, partner change rates were standardized to the GPC on HIV status, age and gender (Details, Appendix S2).

To estimate 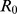, the “effective” mean partner turnover rate is often used as opposed to the arithmetic mean. The effective mean, denoted here, by 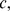 is calculated from the arithmetic mean 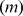 and variance (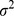) of the distribution of the number of new partners per unit time:



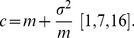
(1)


Anderson and colleagues provide details of Equation 1 [17].

The implication of Equation 1 is that if the variance is high then 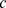 will be significantly larger than 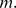 According to May and Anderson [16] and Anderson and May [18], this indicates that the core group plays a disproportionate role in the maintenance and transmission of infection. These authors warn that using 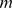 as opposed to 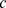 in calculating 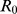 could result in a serious underestimate of 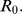 Therefore in this work the effective mean partner change rate was used for all methods.

### Mixing strategies

For scenarios assuming more than one sexual activity class, defined by partner change rate, we considered two types of mixing: assortative and proportionate. In assortative, all partnerships are formed between partners in the same sexual activity class. Under proportionate mixing, the probability that a partner is selected is proportional to the partner change rate of the prospective partner.

R_0_ estimation. Let 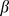 be the probability that an infected individual will infect a susceptible partner over the duration of their relationship, 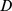 the average duration of infectiousness (years) and 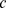
the average number of new partners acquired by an individual per year. The following methods were used to estimate *R_0_* in this work. We used the numbers indicated against each method to reference the method in subsequent sections.

1. The basic method is the product of 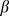, c and D [1].
2. The basic method but with varying 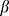 by HIV stage [5], [19]. This includes two scenarios:
  - Varying 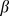 by HIV stage
  - Varying 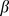 by HIV stage and including probability of survival to a given HIV stage.
3. A method with two sexual activity classes [7], proportionate and assortative mixing assumed, ignoring gender and assuming fixed 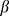 for the entire infectiousness period.
4. Adding to (3) by taking gender into account and assuming:
  - same 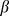 for male and female
  - different 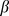 by gender
5. The same as (4) but with three sexual activity classes [7].
6. The basic method with different transmission probabilities by gender fixed for the entire infectiousness period.

Method 1 assumes that the probability that a partner is infected is equal to the proportion of partnerships on offer that are from infected people. Methods 1 to 3 ignore gender while the rest are restricted to heterosexual partnerships. In all methods except 2ii, it was assumed that everybody who gets infected lives through the entire duration of infection i.e. mean of 11 years [11]. For methods with HIV staging, four stages were assumed: the initial high viraemic stage (mean five months), the asymptomatic stage (mean 8.5 years), the pre-AIDS stage (mean 1.66 years), and AIDS (mean five months) (Details, Appendix S3).

### Assessment of partner change rate threshold

To determine the minimum partner change rate that is required to attain 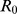 less than one, each 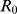 expression was set to less than one (i.e. 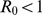) and the expression solved for partner change rate. For instance from method 1,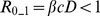 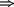 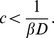 Expressions for the remaining methods are presented in Table S2.

### Effect of reductions of partner change rate on 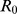


We assessed the effect of partner change rate reductions on 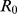. Using method 5, with three sexual activity classes and same 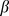 by gender, we determined the impact on 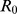 of different reductions in the 2002–2008 average partner change rate within different classes.

## Results

**Figure 1:**
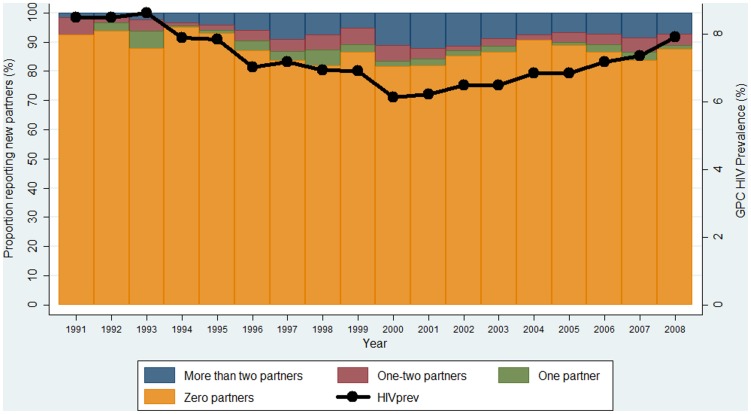
Distribution of annual partner turnover in the rural clinical cohort and HIV prevalence in the general population cohort.

The annual reported number of new partners ranged between 0–8 (1991–1996) and 0–80 (1997–2008) with a notable increase in the reported number of new partners from 1997 (Figure 1). The highest reported number of new partners in a year among males was 80, while that among females was 16. However, majority (82%–95%) of respondents each year reported zero new partners. On average, 90% (Females: 89%–99%; males: 78%–95%) of respondents reported zero to one new partner, 3% (Females: 0%–7%; males: 2%–7%) reported over one to two new partners and 7% (Females: 1%–7%; males: 2%–18%) reported over two new partners per year.

**Figure 2:**
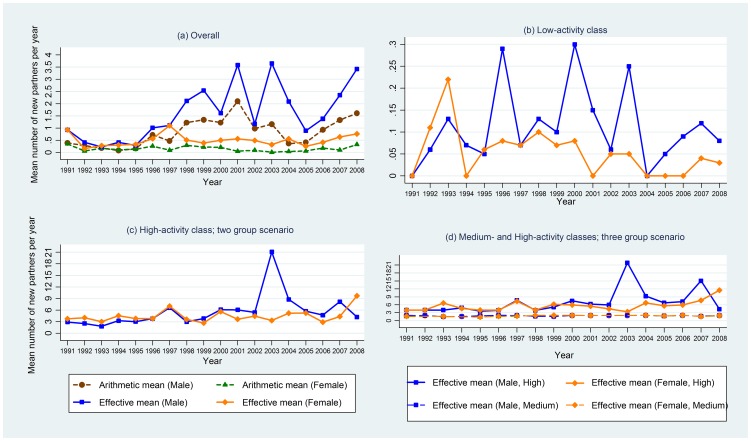
Partner change rates per year; by sexual activity class and gender.

The arithmetic and “effective” mean partner change rate varied by year and gender; especially for the males (Figure 2a). The effective mean partner change rate ranged between 0–0.3 (low-activity), 1.3–2 (medium-activity) and 3.3–21.9 (high-activity class). For years with high variability in reported number of new partners, the effective means were much higher than the means for years with low variability (Figure 2b–d).

### 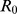 estimates using effective mean partner change rate

The basic method (method 1) yielded 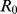 estimates ranging between 0.45–6.34. 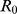 estimates from 1992–1995 were below one. After 1995, 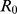 estimates were above one and reached 6.34 in 2003. Estimates from method 2 had a similar trend to estimates from method 1 but were lower. From method 3 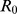 estimates where proportionate mixing was assumed ranged between 2.12–5.85 (1991–1996) and 4.35–33.75 (1997–2008). Under 100% assortative mixing, in the high sexual activity class, 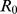 estimates ranged between 4.18–6.69 (1991–1996) and 5.43–36.75 (1997–2008) (Table 1).

**Table 1:** R_0_ estimates from methods 1, 2, 3, 4 and 6.

	Methods 1, 2, 6				Method 3			Method 4: same β by gender			Method 4: different β by gender		
	No sexual activity classes:- R_0_1_:Basic formula_;_ R_0_2_: Basic formula with varying β by HIV stage_;_ R_0_6_:Heterosexual partnerships with different β by gender				Two sexual activity classes but ignoring gender; fixed β: R_0_3_			Heterosexual partnerships:- Two sexual activity classes: R_0_4_			Heterosexual partnerships:- Two sexual activity classes: R_0_4_		
						Assortative mixing			Assortative mixing			Assortative mixing	
Year	R_0_1_	R_0_2i_	R_0_2ii_	R_0_6_	Proportionate mixing	Low	High	Proportionate mixing	Low	High	Proportionate mixing	Low	High
1991	1.64	1.41	1.37	1.54	5.85	-*	5.85	5.8	-	5.8	5.46	-	5.46
1992	0.65	0.56	0.54	0.43	2.31	0.17	4.59	2.72	0.14	5.6	2.56	0.14	5.28
1993	0.45	0.39	0.37	0.42	2.12	0.32	4.18	2.14	0.29	4.13	2.02	0.28	3.89
1994	0.61	0.52	0.51	0.58	5.2	0.13	7.12	5.72	-	6.81	5.39	-	6.42
1995	0.54	0.46	0.45	0.51	4.74	0.1	5.97	4.74	0.1	5.97	4.47	0.1	5.63
1996	1.55	1.34	1.3	1.25	4.4	0.4	6.69	4.86	0.26	6.69	4.58	0.25	6.31
1997	1.94	1.67	1.62	1.83	11.06	0.12	11.81	11.22	0.12	12.03	10.58	0.12	11.34
1998	3.13	2.69	2.61	1.73	4.35	0.2	5.43	4.62	0.21	5.74	4.35	0.2	5.41
1999	3.88	3.35	3.24	1.65	5.51	0.13	6.44	4.64	0.15	5.64	4.37	0.14	5.32
2000	2.52	2.17	2.1	1.48	8.61	0.53	10.62	9.06	0.27	10.34	8.55	0.25	9.75
2001	6.16	5.31	5.15	2.35	9.39	0.26	10.57	7.95	-	8.27	7.49	-	7.79
2002	1.94	1.67	1.62	1.26	8.72	0.1	9.34	7.91	0.09	8.62	7.45	0.09	8.13
2003	6.34	5.47	5.3	1.79	33.75	0.43	36.75	11.21	0.19	14.75	10.57	0.18	13.9
2004	3.38	2.91	2.82	1.79	14.74	-	14.74	11.89	-	11.89	11.21	-	11.21
2005	1.37	1.18	1.15	0.78	9.18	0.1	9.96	9.44	-	9.69	8.9	-	9.14
2006	2.04	1.76	1.71	1.25	6.69	0.16	7.8	6.18	-	6.51	5.83	-	6.14
2007	3.61	3.11	3.02	2.02	12.84	0.17	13.84	9.74	0.12	10.49	9.19	0.12	9.89
2008	4.99	4.3	4.17	2.68	8.17	0.13	9.09	10.5	0.09	11.29	9.9	0.09	10.64

“-” implies R_0_ could not be estimated because, for one or both genders, there were no individuals falling in the sexual activity class.

Methods 4 and 5 (same 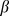 by gender) yielded 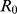 estimates between 2.14–11.89 and 3.06–15.93 for proportionate mixing under scenarios of two and three sexual activity classes respectively. Under assortative mixing, 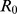 estimates among the high-activity class were 4.13–14.75 (two activity classes - method 4; Table 1) and 6.64–18.91 (three activity classes - method 5; Table 2).

**Table 2:** R_0_ estimates from method 5, with three sexual activity classes.

	R_0_5_: same β by gender				R_0_5_: different β by gender			
		Assortative mixing				Assortative mixing		
Year	Proportionate mixing	Low	Medium	High	Proportionate mixing	Low	Medium	High
1991	4.67	-*	3.15	7.04	4.41	-	2.97	6.63
1992	3.06	0.14	-	7.04	2.88	0.14	-	6.63
1993	3.75	0.29	2.73	9.05	3.53	0.28	2.58	8.53
1994	6.59	-	-	8.29	6.22	-	-	7.81
1995	4.59	0.1	2.77	6.64	4.33	0.1	2.61	6.26
1996	4.23	0.26	3.19	6.96	3.99	0.25	3.01	6.56
1997	11.32	0.12	3.18	13.18	10.67	0.12	3	12.42
1998	4.78	0.21	3.22	6.89	4.5	0.2	3.03	6.49
1999	6.84	0.15	3.12	9.86	6.45	0.14	2.94	9.3
2000	9.2	0.27	3.5	11.71	8.67	0.25	3.3	11.04
2001	8.94	-	3.44	10.29	8.43	-	3.24	9.7
2002	8.1	0.09	-	9.04	7.64	0.09	-	8.52
2003	11.2	0.19	-	15.02	10.56	0.18	-	14.16
2004	13.05	-	3.52	13.85	12.3	-	3.32	13.06
2005	9.47	-	3.14	10.8	8.93	-	2.96	10.18
2006	10.04	-	3.5	11.52	9.47	-	3.3	10.87
2007	15.93	0.12	2.83	18.91	15.02	0.12	2.67	17.83
2008	9.97	0.09	3.44	12.38	9.4	0.09	3.24	11.68

“-” implies R_0_ could not be estimated because, for one or both genders, there were no individuals falling in the sexual activity class.


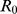
 estimates from method 6 ranged from 0.42–0.58 (1992–1996) and 0.78–2.68 (1997–2008) with 0.78 in 2005, the only year after 1996 in which men had an estimated annual mean partner turnover below one (Table 1).

### Assessment of partner change rate threshold to reduce 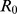 below one

Despite the markedly different 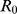
s, the estimated threshold of the partner change rate, i.e., the value required to bring 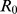 <1, was estimated to be at or near 0.57 for all methods (Table 3, Table S2). The percent reductions in the partner change rate required to bring 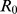 below one are presented in Table 3, column 6. Using an average effective mean partner change rate over the years 2002–2008, methods that assume proportionate mixing among sexual activity classes indicated that, to bring 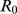 below one, females would have to reduce their partner acquisition rate by 88% and 89% for the two and three class scenarios respectively. For the males, a reduction of 93% and 94% in the case of two and three activity classes, respectively, is required. If 100% assortative mixing among sexual activity classes is assumed, in the high-activity class, females would be required to reduce their partner acquisition rate by 89% and 91% (two and three class scenarios) while the males would be required to reduce their acquisition rate by 93% and 94% (two and three class scenarios).

**Table 3:** 2002–2008 average observed and threshold partner change rates, calculated R_0_ and % reduction in observed partner change rate required to attain R_0_ < 1.

Method	Class	Threshold rate	2002–2008 average observed rate	R_0_	% reduction in observed rate required to attain R_0_ < 1
1		0.57	1.92	3.38	70%
2i		0.66	1.92	2.92	66%
2ii		0.67	1.92	2.83	65%
3 proportionate		0.57	7.61	13.39	93%
3 assortative	Low	0.57	0.09	0.16	NA*
	High		8.25	14.5	93%
4 proportionate	Male	0.57	7.82	10.53	93%
	Female		4.58		88%
4 assortative	Male Low	0.57	0.09	0.12	NA*
	Female Low		0.02		NA*
	Male High		8.31	11.38	93%
	Female High		5.04		89%
5 proportionate	Male	0.57	8.95	12.22	94%
	Female		5.3		89%
5 assortative	Male Low	0.57	0.09	0.12	NA*
	Female Low		0.02		NA*
	Male Medium		1.91	3.31	70%
	Female Medium		1.85		69%
	Male High		10.05	14.17	94%
	Female High		6.45		91%
6	Male	0.43	2.14	1.69	80%
	Female	0.85	0.49		NA*

Per cent reduction not calculated since the observed rate was below the threshold.

### Effect of reductions in partner change rate on 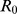


**Figure 3:**
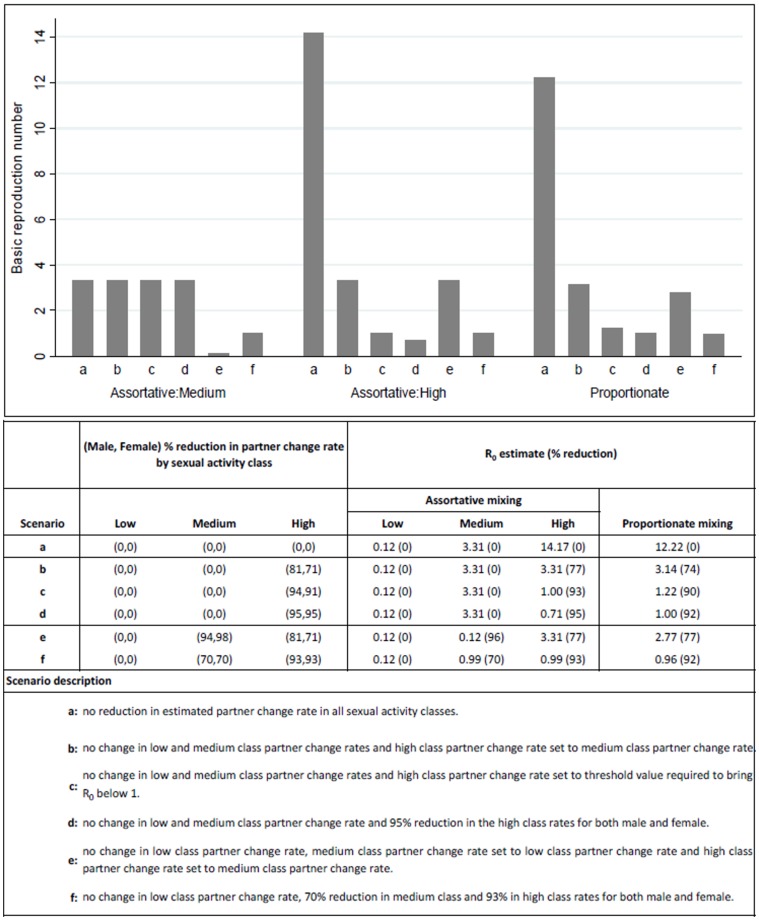
Effect of reduction in the 2002–2008 average effective mean partner change rate on R_0_ estimates from method 5.

We calculated the 2002–2008 average effective mean partner change rate and used it in method 5, of three activity classes and assuming same 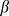 by gender, to assess the effect of, several of, its reductions on 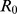. Maintaining the low-activity class partner change rate but reducing the medium class rate to low-activity rate (reduction of 94% (males) and 98% (females)) and reducing high class rate to medium-activity level (reduction of 81% (males) and 71% (females)) would result in 77% (proportionate mixing), 96% (medium class) and 77% (high class) reductions in 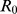 estimates. However, the resulting proportionate and high-activity class 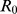 estimates would still be above one (Figure 3, scenario e). On the other hand, maintaining low and medium class partner change rates but reducing the high class rate to the threshold level required to bring 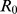 below one, i.e., by 94% (male) and 91% (female) resulted in a 90% (proportionate) and 93% (high-activity class) reduction in 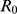 to values of 1.22 and 1.00 respectively (Figure 3, scenario c). If instead the high class partner turnover was reduced by at least 95% for both genders, overall 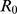 proportionate estimate would be below one (Figure 3, scenario d).

## Discussion

To eradicate the HIV/AIDS epidemic in this population, the 8% of the adult population who, on average, reported more than two new partners per year will have to reduce their partner acquisition rate drastically. The low risk takers could maintain their sexual behaviour; the 3% population reporting more than one to two new partners per year would have to reduce their partner acquisition rate by at least 70%; while the 8% high risk population reporting more than two new partners per year would have to reduce the partner acquisition rate by at least 93%. On the other hand, if the 8% high risk takers were the only ones to reduce partner acquisition, a reduction of 95% would be required to eradicate the epidemic.

All methods assuming proportionate mixing yielded 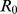 above one. Under assortative mixing, estimates for the medium- and high-activity classes yielded 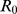 estimates above one, while estimates in the lowest sex activity class were below one. The methods using varying 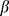 by stage (methods 2i and 2ii) yielded lower estimates than those where staging was not considered. Method 3, which ignored gender, produced 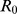 estimates comparable to methods that assumed exclusively heterosexual partnerships.

For methods 4 and 5, under proportionate mixing, estimates from the two class scenario were in general lower than those under the three class scenario. A similar trend was observed in the high-activity class (assortative mixing). Method 5 where three sexual activity classes were used demonstrated the contribution of a small proportion of high risk individuals on the epidemic. Comparison of 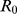 estimates in the high-activity class (two class scenario) to those in the medium- and high-activity classes (three class scenario) indicated that merging the medium- and high-activity classes in the two case scenario, masked the impact of the high-activity class. We observed estimates in the range of 4.13–14.75 (two class scenario) compared to 6.64–18.91 (three classes scenario) using same 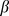 by gender.

Estimates from method 6, with all individuals belonging to one sexual activity class and varying 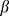 by gender, were lower than estimates from the basic method but with similar trend.

In general, estimates assuming different transmission probabilities by gender were lower than those where a non-gender specific transmission probability was used. The differences arose because we estimated the non-gender specific transmission probability as an arithmetic mean of the gender specific transmission probabilities. 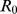 formulae using gender specific transmission probabilities implied that we were essentially using a geometric mean of the two probabilities and this is lower than the arithmetic mean. If the non-gender specific transmission probability had been estimated using a geometric mean as opposed to an arithmetic mean, the resulting 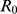 estimates would have been identical to those from methods where we assumed gender specific transmission probabilities. Thus attaining equal 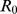 estimates using non-gender and gender specific transmission probabilities would require that the non-gender specific 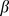 is equal to 0.1508, the square root of the product of the gender specific 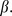


The similarity in the estimates of the partner change rate threshold for the various methods (all around 0.57 partners per year) was because they all depended on the relationship 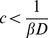
(Table S2). These results imply that to be able to bring 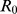 below one, the overall mean partner change rate should not be more than 0.57 partners per year, i.e., approximately not more than 1 partner every two years. To lower the effective partner change rate would require either a reduced mean partner turnover or a reduced variance in partner turnover which could be achieved by focussing on reducing the partner turnover among those with very high partner turnover while allowing those with low partner turnover to remain the same, or a combination of both.

This work has some limitations. From the empirical data we noted that although the partner change rates in the two genders were in comparable range for most years, during 2003, 2007 and 2008, there was a wide gap. This highlights the drawback of using reported sexual behaviour data. Also in 1992, 1994, 2002 and 2003, females did not report more than one to two new partners in a year. This meant that 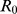 in the medium sexual activity class (three class scenario) under 100% assortative mixing was zero. However, we are aware that the assumptions of 100% assortative mixing and full proportionate mixing are on extreme ends. 100% assortativity provides the plausible minimum and maximum R_0_ estimates from the low-activity and high-activity classes respectively; while assessing 100% proportionate mixing does the opposite. It provides the maximum and minimum R_0_ estimates from the low-activity and high-activity classes, respectively. In reality, mixing is somewhere between proportionate and 100% assortative [20], [21]. As such, these scenarios are used to provide minimum and maximum R_0_ estimates.

Another problem that could arise is the over estimation of the effective mean partner change rate in certain sexual activity groups. Although the sample sizes in the RCC are not small, ranging from 121 to 352 per year; once we have stratified by HIV status, age group, and gender, the numbers can become small. This is likely to impact on the precision of the estimated effective mean.

## Conclusion

This work demonstrated that there can be differences in 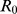 estimates by method of estimation. Estimates depend on the assumptions that one makes especially regarding to mixing. For instance ignoring existence of variation in sexual behaviour and not examining how people mix may lead to underestimation of 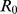. We have also shown that the amount of reduction in partner turnover necessary in our population in order to bring 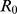 under one would be very high, so achieving HIV eradication through this strategy alone may be infeasible. Complementary HIV prevention strategies such as male circumcision and HIV treatment services therefore need rapid scale up.

## Supporting Information

Table S1 **Parameter estimates for duration and transmission probability by HIV stage, based on empirical literature by Wawer et al.**
(DOC)

Table S2
**Projection of partner change rate required to bring** 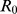 **below one.**
(DOC)

Appendix S1 **Estimation of the background mortality rate.**
(DOC)

Appendix S2 **Standardisation of the mean and variance of new partners.**
(DOC)

Appendix S3 **Methods used to estimate** ***R_0._***
(DOC)

## References

[1] Anderson RM, May RM (1988) Epidemiological parameters of HIV transmission. Nature 333: 514–519. 3374601 10.1038/333514a0

[2] Dietz K (1993) The estimation of the basic reproduction number for infectious diseases. Stat Methods Med Res 2: 23–41. 8261248 10.1177/096228029300200103

[3] Jacquez JA, O'Neill P (1991) Reproduction Numbers and Thresholds in Stochastic Epidemic Models I. Homogeneous Populations. Mathematical Biosciences 107: 161–186. 1806112 10.1016/0025-5564(91)90003-2

[4] Dietz K, Heesterbeek JAP, Tudor DW (1993) The Basic Reproduction Ratio for Sexually Transmitted Diseases. Part 2. Effects of Variable HIV Infectivity. Mathematical Biosciences 117: 35–47. 8400583 10.1016/0025-5564(93)90016-4

[5] Hyman JM, Li J (2000) An intuitive formulation for the reproductive number for the spread of diseases in heterogeneous populations. Mathematical Biosciences 167: 65–86. 10942787 10.1016/s0025-5564(00)00025-0

[6] Diekmann O, Dietz K, Heesterbeek JAP (1991) The Basic Reproduction Ratio for Sexually Transmitted Diseases: I. Theoretical Considerations. Mathematical Biosciences 107: 325–339. 1806121 10.1016/0025-5564(91)90012-8

[7] Diekmann O, Heesterbeek JAP, Metz JAJ (1990) On the definition and the computation of the basic reproduction ratio R_0_ in models for infectious diseases in heterogeneous populations. J Math Bio 28: 365–382. 2117040 10.1007/BF00178324

[8] Seeley J, Wagner U, Mulemwa J, Kengeya-Kayondo J, Mulder D (1991) The development of a community-based HIV/AIDS counselling service in a rural area in Uganda. AIDS Care 3: 207–217. 1878404 10.1080/09540129108253064

[9] Morgan D, Malamba SS, Maude GH, Okongo MJ, Wagner HU, et al (1997) An HIV-1 natural history cohort and survival times in rural Uganda. AIDS 11: 633–650. 9108945 10.1097/00002030-199705000-00011

[10] Kazooba P, Kasamba I, Baisley K, Mayanja BN, Maher D (2012) Access to, and uptake of, antiretroviral therapy in a developing country with high HIV prevalence: a population-based cohort study in rural Uganda, 2004-2008. Tropical Medicine and International Health 17: E49–E57. 22943379 10.1111/j.1365-3156.2012.02942.x PMC3443381

[11] Wawer MJ, Gray RH, Sewankambo NK, Serwadda D, Li X, et al (2005) Rates of HIV-1 Transmission per Coital Act, by Stage of HIV-1 Infection, in Rakai, Uganda. JID 191: 1403–1409. 15809897 10.1086/429411

[12] Van der Paal L, Shafer LA, Todd J, Mayanja BN, Whitworth JA, et al (2007) HIV-1 disease progression and mortality before the introduction of highly active antiretroviral therapy in rural Uganda. AIDS 21: S21–S29. 10.1097/01.aids.0000299407.52399.05 18032935

[13] Morgan D, Malamba S, Orem J, Mayanja B, Okongo M, et al (2000) Survival by AIDS defining condition in rural Uganda. Sex Transm Infect 76: 193–197. 10961197 10.1136/sti.76.3.193 PMC1744150

[14] Todd J, Glynn JR, Marston M, Lutalo T, Biraro S, et al (2007) Time from HIV seroconversion to death: a collaborative analysis of eight studies in six low and middle-income countries before highly active antiretroviral therapy. AIDS 21: S55–S63. 10.1097/01.aids.0000299411.75269.e8 PMC5784803 18032940

[15] INDEPTH-Network (2004) INDEPTH Model Life Tables for Sub-Saharan Africa; Ngom P, Bawah AA, editors: Ashgate.

[16] May RM, Anderson RM (1987) Transmission dynamics of HIV infection. Nature 326: 137–142. 3821890 10.1038/326137a0

[17] Anderson RM, Medley GF, May RM, Johnson AM (1986) A preliminary Study of the Transmission Dynamics of the Human Immunodeficiency Virus (HIV), the Causative Agent of AIDS. IMA J of Math Appl in Med & Bio 3: 229–263. 3453839 10.1093/imammb/3.4.229

[18] Anderson RM, May RM (1991) Infectious Diseases of Humans: Dynamics and Control: Oxford University Press.

[19] Leynaert B, Downs AM, de Vincenzi I (1998) Heterosexual Transmission of Human Immunodeficiency Virus Variability of Infectivity throughout the Course of Infection.European Study Group on Hetrosexual Transmission of HIV. Am J Epidemiol 148: 88–96. 9663408 10.1093/oxfordjournals.aje.a009564

[20] Gupta S, Anderson RM, May RM (1989) Networks of sexual contacts: implications for the pattern of spread of HIV. AIDS 3: 807–817. 2517202

[21] Garnett GP, Hughes JP, Anderson RM, Stoner BP, Aral SO, et al (1996) Sexual mixing patterns of patients attending sexually transmitted diseases clinics. Sexually Transmitted Diseases 23: 248–257. 8724517 10.1097/00007435-199605000-00015

