# Supplementary material for: Estimation of the HIV Basic Reproduction Number in Rural South West Uganda: 1991–2008
Source: PLoS One. 2014 Jan 3;9(1):e83778. doi: 10.1371/journal.pone.0083778 (PMC3880255; doi:10.1371/journal.pone.0083778)
Supplement: Table S1 — Parameter estimates for duration and transmission probability by HIV stage, based on empirical literature by Wawer et al. (DOC) [file pone.0083778.s001.doc]

**Supporting information Table S1: Parameter estimates for duration and transmission probability by HIV stage, based on empirical literature by Wawer et al**

| **Stage** | **1** | **2** | **3** | **4** | **All stages** |
| --- | --- | --- | --- | --- | --- |
| Duration(years) | 0.4 | 8.5 | 1.7 | 0.4 | 11 |
| Transmission probability/ act | 0.0082 | 0.0009 | 0.0038 | 0 | 0.0016 |
| Transmission probability/ partnership (average for both  M->F and F->M) | 0.4097 | 0.1024 | 0.2853 | 0 | 0.1599 |
| Transmission probability/ partnership ( M->F) | 0.5463 | 0.1365 | 0.3804 | 0 | 0.2132 |
| Transmission probability/ partnership ( F->M) | 0.2731 | 0.0683 | 0.1902 | 0 | 0.1066 |

**M->F: transmission from Male to female; F->M: transmission from Female to male**

**References**

1. Wawer MJ, Gray RH, Sewankambo NK, Serwadda D, Li X, et al. (2005) Rates of HIV-1 Transmission per Coital Act, by Stage of HIV-1 Infection, in Rakai, Uganda. JID 191: 1403-1409.
