# Supplementary material for: Estimation of the HIV Basic Reproduction Number in Rural South West Uganda: 1991–2008
Source: PLoS One. 2014 Jan 3;9(1):e83778. doi: 10.1371/journal.pone.0083778 (PMC3880255; doi:10.1371/journal.pone.0083778)
Supplement: Table S2 — Projection of partner change rate required to bring below one. (DOC) [file pone.0083778.s002.doc]

**Supporting information Table S2: Projection of partner change rate required to bring $R_{0}$below one**

| **Method** | **Formula*** | **Partner change rate for** $R_{0}<1$ | | **Result** |
| --- | --- | --- | --- | --- |
| 1 | $R_{0\_1}=\beta cD$ | $c<\frac{1}{\beta D}$ |  | $c<0.569$ |
| 2i | $R_{0\_2i}=c\sum_{s=1}^{4} \beta_{s}D_{s}$ | $c<\frac{1}{\sum_{s=1}^{4} \beta_{s}D_{s}}$ |  | $c<0.659$ |
| 2ii | $R_{0\_2ii}=c\sum_{s=1}^{4} p_{s}\beta_{s}D_{s}$ | $c<\frac{1}{\sum_{s=1}^{4} p_{s}\beta_{s}D_{s}}$ |  | $c<0.667$ |
| 3 proportionate |  | $\kappa<\frac{1}{D\beta}$ | $\kappa=\sum_{a=1}^{2} f_{a}c_{a}$ | $\kappa<0.569$ |
| 3 assortative | $\text{Low:}R_{0\_3}=\beta c_{1}D$ $\text{High:}R_{0\_3}=\beta c_{2}D$ | $c_{1}<\frac{1}{\beta D}$ $c_{2}<\frac{1}{\beta D}$ |  | $c_{1}<0.569$ $c_{2}<0.569$ |
| 4 proportionate |  | $\kappa_{1}\kappa_{2}<\frac{1}{D^{2}\beta^{2}}$ | $\kappa_{g}=\sum_{a=1}^{2} f_{g,a}c_{g,a}$ | $\kappa_{1}\kappa_{2}<0.323$ |
|  |  | $\kappa_{1}\kappa_{2}<\frac{1}{D^{2}\beta_{1}\beta_{2}}$ |  | $\kappa_{1}\kappa_{2}<0.364$ |
| 4 assortative | $\text{Low:}R_{0\_4i}=D\beta\sqrt{c_{1,1}c_{2,1}}$ $\text{High:}R_{0\_4i}=D\beta\sqrt{c_{1,2}c_{2,2}}$ | $c_{1,1}c_{2,1}<\frac{1}{D^{2}\beta^{2}}$ $c_{1,2}c_{2,2}<\frac{1}{D^{2}\beta^{2}}$ |  | $c_{1,1}c_{2,1}<0.323$ $c_{1,2}c_{2,2}<0.323$ |
|  | $\text{High:}R_{0\_4ii}=D\sqrt{\beta_{1}c_{1,2}\beta_{2}c_{2,2}}$ | $c_{1,1}c_{2,1}<\frac{1}{D^{2}\beta_{1}\beta_{2}}$ $c_{1,2}c_{2,2}<\frac{1}{D^{2}\beta_{1}\beta_{2}}$ |  | $c_{1,1}c_{2,1}<0.364$ $c_{1,2}c_{2,2}<0.364$ |
| 5 proportionate |  | $\kappa_{1}\kappa_{2}<\frac{1}{D^{2}\beta^{2}}$ | $\kappa_{g}=\sum_{a=1}^{3} f_{g,a}c_{g,a}$ | $\kappa_{1}\kappa_{2}<0.323$ |
|  |  | $\kappa_{1}\kappa_{2}<\frac{1}{D^{2}\beta_{1}\beta_{2}}$ |  | $\kappa_{1}\kappa_{2}<0.364$ |
| 5 assortative |  | $c_{1,1}c_{2,1}<\frac{1}{D^{2}\beta^{2}}$ $c_{1,2}c_{2,2}<\frac{1}{D^{2}\beta^{2}}$ $c_{1,3}c_{2,3}<\frac{1}{D^{2}\beta^{2}}$ |  | $c_{1,1}c_{2,1}<0.323$ $c_{1,2}c_{2,2}<0.323$ $c_{1,3}c_{2,3}<0.323$ |
|  | $\text{Medium:}R_{0\_5ii}=D\sqrt{\beta_{1}c_{1,2}\beta_{2}c_{2,2}}$ | $c_{1,1}c_{2,1}<\frac{1}{D^{2}\beta_{1}\beta_{2}}$ $c_{1,2}c_{2,2}<\frac{1}{D^{2}\beta_{1}\beta_{2}}$ $c_{1,3}c_{2,3}<\frac{1}{D^{2}\beta_{1}\beta_{2}}$ |  | $c_{1,1}c_{2,1}<0.364$ $c_{1,2}c_{2,2}<0.364$ $c_{1,3}c_{2,3}<0.364$ |
| 6 | $R_{0\_6}=D\sqrt{\beta_{1}c_{1}\beta_{2}c_{2}}$ | $c_{2}<\frac{1}{D\beta_{2}}$ $c_{1}<\frac{1}{D\beta_{1}}$ |  | $\begin{matrix} c_{1}<0.426 \\ c_{2}<0.853 \end{matrix}$ |

***For methods 4 and 5; subscript “*i*” implies same $\beta$by gender and “*ii*”: different $\beta$ by gender**
