# Supplementary material for: Estimation of the HIV Basic Reproduction Number in Rural South West Uganda: 1991–2008
Source: PLoS One. 2014 Jan 3;9(1):e83778. doi: 10.1371/journal.pone.0083778 (PMC3880255; doi:10.1371/journal.pone.0083778)
Supplement: Appendix S1 — Estimation of the background mortality rate. (DOC) [file pone.0083778.s003.doc]

**Supporting information Appendix S1: Estimation of the background mortality rate**

Using the Brass demographic life table, the background mortality (not due to HIV) between 15 and 60 years of age (age range assumed to be sexually active) was estimated. The best fit to the HIV negative population background mortality in the GPC data yielded an estimate of 0.1645 over the 45 years which is equivalent to a mortality rate of

$\mu=\int_{0}^{0.0222} 0.1645e^{-0.1645x}dx=0.00365$per year.

Where $0.0222=\frac{1}{45}.$

**References**

1. INDEPTH-Network (2004) INDEPTH Model Life Tables for Sub-Saharan Africa; Ngom P, Bawah AA, editors: Ashgate.
