# Supplementary material for: Estimation of the HIV Basic Reproduction Number in Rural South West Uganda: 1991–2008
Source: PLoS One. 2014 Jan 3;9(1):e83778. doi: 10.1371/journal.pone.0083778 (PMC3880255; doi:10.1371/journal.pone.0083778)
Supplement: Appendix S2 — Standardisation of the mean and variance of new partners. (DOC) [file pone.0083778.s004.doc]

**Supporting information Appendix S2: Standardisation of the mean and variance of new partners**

Below we review standardising by describing the method of standardising the mean number of new partners from the RCC to the GPC based on HIV status, age and gender.

Note: Dividing the age in four categories gives 16 combinations of HIV status, gender and age (2 × 2 × 4).

Let $a$be the activity class; with a = 1 (low), 2(medium), 3(high).

$k$ indicate the category of the characteristics on which the standardisation is based

(e.g. $k=1\Rightarrow$Male, HIV-, 13-24 years; k ranges from 1 to 16)

$p_{ak}$ = the proportion of respondents in category$k$and sexual activity $a$ in the RCC

$N_{k}$ = the population of category$k$in the GPC in a given year

$\upsilon_{ak}$ = un-standardised arithmetic mean in sexual activity *a* of category *k.*

then the standardised mean (m) and variance ($\sigma^{2}$) are given by

$m=\sum_{k} \sum_{a} \upsilon_{ak}\frac{p_{ak}N_{k}}{\sum_{k} \sum_{a} p_{ak}N_{k}}$ and $\sigma^{2}=\sum_{k} \sum_{a} [\upsilon_{ak}-m]^{2}\frac{p_{ak}N_{k}}{\sum_{k} \sum_{a} p_{ak}N_{k}}.$
