# Supplementary material for: Estimation of the HIV Basic Reproduction Number in Rural South West Uganda: 1991–2008
Source: PLoS One. 2014 Jan 3;9(1):e83778. doi: 10.1371/journal.pone.0083778 (PMC3880255; doi:10.1371/journal.pone.0083778)
Supplement: Appendix S3 — Methods used to estimate R0. (DOC) [file pone.0083778.s005.doc]

**Supporting information Appendix S3: Methods used to estimate *R_0_***

### Calculation of *R_0_* using the basic method of Anderson and May

Anderson and May stated a basic expression of calculating$R_{0}$as simply the product of transmission probability ($\beta$), partner change rate and infectiousness duration *i.e.*

$R_{0}=\beta cD,$ (denoted by$R_{0\_1}$) **.** (2)

The implicit assumptions made here are that mixing between individuals is proportionate to the number of partnerships on offer,$\beta$and$D$are fixed for any infectious individual and also that$\beta$is fixed, regardless of partnership duration .

### The basic method but with varying transmission probability and duration of infectiousness by HIV stage

Here two methods were considered: one assumes varying transmission probability by infection stage. The other by Hyman and Li assumes varying transmission probability by infection stage and includes the probability of an infected individual surviving to a given stage.

### 2.1 *R_0_* assuming varying$\beta$and *D* by HIV stage

The assumption here was that $c$does not vary with stage, but$\beta$and$D$do. Equation 2 was therefore modified to give$R_{0}$ (denoted$R_{0\_2i}$) as

$R_{0\_2i}=c\sum_{s=1}^{4} \beta_{s}D_{s}$ (3)

where$\beta_{s}$and $D_{s}$ are, respectively, the transmission probability and infectiousness duration associated with HIV stage$s.$

### 2.2 *R_0_* assuming varying$\beta$and *D* by HIV stage including probability of survival to a given stage

Here it was assumed that not all individuals will survive to the next stage . Therefore Equation 3 was modified to include the probability $(p_{s})$of an infected individual surviving to stage *s.* It was also assumed that $p_{1}=1.$The resulting $R_{0}$(denoted$R_{0\_2ii}$) was given by

$R_{0\_2ii}=c\sum_{s=1}^{4} p_{s}\beta_{s}D_{s}$ (4)

It was assumed that failure to progress to the next stage was due to, only, constant background mortality$\mu$i.e. the time to natural death is exponentially distributed with mean$\frac{1}{\mu}.$Thus the proportion of individuals that progress to the next stage was the proportion that lives to stage *s+1*, given that they were alive at the beginning of stage *s*. This is given by $1-\int_{0}^{Ds} \mu e^{-\mu x}dx=e^{-\mu D_{s}}$and hence

$p_{s}=\prod_{s=1}^{s-1} e^{-\mu D_{s}};s=2,3,4$ and $p_{1}=1.$ (5)

This derivation of$p_{s}$gives similar results as that given by Hyman and Li in which $p_{s}=\prod_{s=1}^{s-1} \frac{\gamma_{s}}{\mu+\gamma_{s}}$**,** where$\gamma_{s}$is the average rate of progression from stage $s$ to stage $s+1$ (equivalent to$\frac{1}{D_{s}}$) and$\mu$is the background mortality rate. Then

$R_{0\_2ii}=c\sum_{s=1}^{4} \frac{\beta_{s}p_{s}}{\mu+\gamma_{s}}$ (6)

where $D_{s}$in Equation 4 is equivalent to $\frac{1}{\mu+\gamma_{s}}.$

Although not presented in this work, if in addition to $\mu$we assume HIV related mortality $\mu_{h_{s}}$in each infection stage, then $R_{0}$ would be given by

$R_{0\_2iii}=c\sum_{s=1}^{4} \frac{\beta_{s}p_{s}}{\mu+\mu_{h_{s}}+\gamma_{s}}.$

### A method with the population split according to two sexual activity classes but ignoring gender

The methods presented so far did not account for sexual activity heterogeneity. This section considered a method where the population was split into two sexual activity classes with 100% proportionate and 100% assortative mixing assumed between the classes, assuming both$\beta$and *D* are fixed for the entire infectiousness period but ignoring gender. In proportionate mixing, the probability that a partner is selected is proportionate to the partner change rate of the prospective partner, while in assortative mixing, all partnerships are formed with people in the same sexual activity class as the index partner.

Sexual activity was defined based on the annual reported number of new partners. First, two sexual activity classes were defined as follows:

1. Low: those who reported 0 or 1 new partner in the year
2. High: those who reported more than one new partner in the year.

Let$p_{i}$be the proportion of the population in class$i$, $c_{i}$ be the partner change rate in class$i$, $f_{i}$ the fraction of partnerships from class $i$, given by$f_{i}=\frac{p_{i}c_{i}}{\sum_{i=1}^{2} p_{i}c_{i}}$, $k_{ij}$the number of new partners formed by individuals from class$j$with those from class $i$, given by $k_{ij}=c_{j}f_{i}$, $R_{ij}$the number of secondary infections from each infected member of class$j$ to class$i$ given by $R_{ij}=\beta k_{ij}D=\beta c_{j}f_{i}D;$ $i,j=1,2,$and$\beta$the transmission probability.

$R_{0}$ (denoted $R_{0\_3}$) was calculated as the largest eigenvalue of the next generation 2 by 2 matrix, **R_2_** whose entries are the$R_{ij}$. A next generation matrix is a matrix that summarises the number of secondary infections in a given category arising from individuals in each of the categories.$R_{0}$was calculated on both the assumption of full (100%) assortative mixing i.e. individuals making partnership only with individuals of the same sexual activity preference (like-with-like) and complete proportionate mixing between activity classes where the likelihood of forming a partnership with an individual depends on the number of partnerships on offer (partner change rate) of the individual.

The next generation matrix **R_2_** is given by

Let $\text{p}_{\text{R}_{2}}\text{(}\lambda\text{)}$be a characteristic polynomial of matrix$\text{R}$ defined by

$p_{R_{2}}(\lambda)=\lambda^{2}-\lambda trace(R_{2})+\det(R_{2}),$

where$\lambda$ is the eigenvalue of $\text{R}_{2}$, trace($\text{R}_{2}$) is the sum of the diagonal elements of

$\text{R}_{2}$= $R_{11}+R_{22}$ and det($\text{R}_{2}$) is the determinant of $\text{R}_{2}$= $R_{11}R_{22}-R_{12}R_{21}$. Values of $\lambda$ are obtained by equating $p_{R_{2}}(\lambda)$to zero. Substituting for the trace and determinant in the characteristic polynomial and equating it to zero yields a quadratic equation in$\lambda$: $\lambda^{2}-\lambda(R_{11}+R_{22})+R_{11}R_{22}-R_{12}R_{21}=0$ with solutions:

$\lambda=\frac{1}{2}\left\{(R_{11}+R_{22})\pm\sqrt{(R_{11}-R_{22})^{2}+4R_{12}R_{21}}\right\}.$ (7)

Substituting $\beta c_{j}f_{i}D$ for the$R_{ij}$in Equation 7 gives$\lambda=0$and$\lambda=\beta D\sum_{i=1}^{2} c_{i}f_{i}.$ $R_{0}$is the largest eigenvalue, therefore $R_{0\_3}=\beta D\sum_{i=1}^{2} c_{i}f_{i}$under the assumption of proportionate mixing.

Under the assumption of 100% assortative mixing, $R_{12}=R_{21}=0,$ $f_{1}=f_{2}=1,$and$R_{0}$is estimated separately for each sexual activity class. In the low activity class,$R_{0\_3}=\beta c_{1}D$and in the high activity class, $R_{0\_3}=\beta c_{2}D.$

### A method with the population split according to two sexual activity classes and taking gender into account

The method addressed in this section involved splitting the population into two sexual activity classes, as in Section 2.2.3, with proportionate and assortative mixing assumed, but taking gender into account such that only heterosexual partnerships are formed and also assuming:

1. same $\beta$ for male and female, $\beta$ fixed for the entire infectiousness period.
2. different $\beta$by gender, but fixed for the entire infectiousness period.

There are four categories namely:

1,1: Male of low sexual activity

1,2: Male of high sexual activity

2,1: Female of low sexual activity

2,2: Female of high sexual activity.

Let$g$denote gender, with $g=1$for male, $g=2$for female and where $g'$ denotes the opposite gender of$g;$and$a$ denotes sexual activity class with $a=1$for low and $a=2$for high. The other parameters in the previous section still retain the same definition but with different subscripts/superscripts to reflect the gender and sexual activity classes for each of the categories. We formulate the next generation 4 by 4 matrix, **R_4_** with entries$R_{g,a}^{g',a}$given by

$R_{g,a}^{g',a}=\beta_{g'}k_{g,a}^{g',a}D;$ $a,g,g'=1,2;$where$k_{g,a}^{g',a}=f_{g,a}c_{g',a}$ and $f_{g,a}=\frac{p_{g,a}c_{g,a}}{\sum_{a=1}^{2} p_{g,a}c_{g,a}}.$

Since we assume heterosexual partnerships, $R_{g,a}^{g,a}=0$ for all $g,a$ such that

$$\text{R}_{4}=\left(\begin{matrix} 0 & 0 & R_{1,1}^{2,1} & R_{1,1}^{2,2} \\ 0 & 0 & R_{1,2}^{2,1} & R_{1,2}^{2,2} \\ R_{2,1}^{1,1} & R_{2,1}^{1,2} & 0 & 0 \\ R_{2,2}^{1,1} & R_{2,2}^{1,2} & 0 & 0 \end{matrix}\right)$$

Let $\text{R}_{2}^{\text{M}}=\left(\begin{matrix} R_{1,1}^{2,1} & R_{1,1}^{2,2} \\ R_{1,2}^{2,1} & R_{1,2}^{2,2} \end{matrix}\right),$ $\text{R}_{2}^{\text{F}}=\left(\begin{matrix} R_{2,1}^{1,1} & R_{2,1}^{1,2} \\ R_{2,2}^{1,1} & R_{2,2}^{1,2} \end{matrix}\right)$ and $\text{K}_{2}=\text{R}_{2}^{\text{M}}\text{.R}_{2}^{\text{F}}=\left(\begin{matrix} \sum_{a=1}^{2} R_{1,1}^{2,a}R_{2,a}^{1,1} & \sum_{a=1}^{2} R_{1,1}^{2,a}R_{2,a}^{1,2} \\ \sum_{a=1}^{2} R_{1,2}^{2,a}R_{2,a}^{1,1} & \sum_{a=1}^{2} R_{1,2}^{2,a}R_{2,a}^{1,2} \end{matrix}\right)$

$R_{0}$was calculated as the dominant eigenvalue of $\text{R}_{4}$.

From matrix algebra theory , it can be shown that the determinant of a diagonal matrix$\text{R}_{4}$is equal to the determinant of the product of the two sub-matrices in the diagonal matrix, $\text{K}_{2}$. Also it can be shown that the dominant eigenvalue of $\text{R}_{4}$is the square root of the dominant eigenvalue of $\text{K}_{2}.$Thus$R_{0}$can be estimated by setting the characteristic polynomial of $\text{K}_{2}$ to zero and solving for the eigenvalue variable and then taking the square root of the dominant eigenvalue. The characteristic polynomial of $\text{K}_{2}$ is: $\lambda^{2}-\lambda trace(K_{2})+\det(K_{2}).$

The rows and columns of matrix $\text{K}_{2}$are not linearly independent, implying that det($\text{K}_{2}$) is zero . Thus the characteristic polynomial reduces to:$\lambda^{2}-\lambda trace(K_{2}).$ Equating the equation to zero and solving for $\lambda$, yields $\lambda=0$ and $\lambda=trace(K_{2})$ as the solutions. Thus$R_{0}$, the dominant eigenvalue of matrix **R**_4_ is given by

$$\sqrt{trace(K_{2})}=\sqrt{\sum_{a=1}^{2} R_{1,1}^{2,a}R_{2,a}^{1,1}+\sum_{a=1}^{2} R_{1,2}^{2,a}R_{2,a}^{1,2}.}$$

Substituting for the $R_{g,a}^{g',a}$gives

$R_{0}=D\sqrt{\beta_{1}\beta_{2}\left(\sum_{a=1}^{2} f_{1,a}c_{1,a}\right)\left(\sum_{a=1}^{2} f_{2,a}c_{2,a}\right)}$. (8)

Under the assumption of proportionate mixing and same transmission probabilities by gender, $R_{0}$(denoted$R_{0\_4i}$) is

$$R_{0\_4i}=D\beta\sqrt{\left(\sum_{a=1}^{2} f_{1,a}c_{1,a}\right)\left(\sum_{a=1}^{2} f_{2,a}c_{2,a}\right)}$$

and under the assumption of proportionate mixing and different transmission probabilities by gender, $R_{0\_4ii}=D\sqrt{\beta_{1}\beta_{2}\left(\sum_{a=1}^{2} f_{1,a}c_{1,a}\right)\left(\sum_{a=1}^{2} f_{2,a}c_{2,a}\right)}$

Under 100% assortative mixing, $R_{g,a}^{g',a'}=0$ for all $a\neq a'$and $f_{g,a}=1$ for all $g,a.$ Assuming same transmission probability by gender,

$R_{0\_4i}=D\beta\sqrt{c_{1,1}c_{2,1}}\text{ }$and$D\beta\sqrt{c_{1,2}c_{2,2}}$ for the low and high activity classes respectively.

Assuming different transmission probability by gender,

$R_{0\_4ii}=D\sqrt{\beta_{1}\beta_{2}c_{1,1}c_{2,1}}\text{ }$and$D\sqrt{\beta_{1}\beta_{2}c_{1,2}c_{2,2}}$for the low and high activity classes respectively.

### A method with the population split into three sexual activity classes and taking gender into account

To further assess the effect of heterogeneity in sexual activity on *R_0_*, three sexual activity classes were defined as:

Low: individuals who reported 0 or 1 new partner in the year,

Medium: those who reported more than one to two new partners in the year,

High: those who reported more than two new partners in the year.

We assumed that only heterosexual partnerships are formed and

1. same $\beta$ for male and female, $\beta$ for the entire infectiousness period.
2. Different $\beta$by gender, but fixed for the entire infectiousness period.

There are six categories denoted as follows

1,1: Male of low sexual activity

1,2: Male of medium sexual activity

1,3: Male of high sexual activity

2,1: Female of low sexual activity

2,2: Female of medium sexual activity

2,3: Female of high sexual activity

We assume that only heterosexual partnerships are formed. Following the same formulation as in the previous section, the next generation matrix summarising the number of secondary infections in a given category arising from individuals in each of the categories is given by

$$\text{R}_{6}=\left(\begin{matrix} 0 & 0 & 0 & R_{1,1}^{2,1} & R_{1,1}^{2,2} & R_{1,1}^{2,3} \\ 0 & 0 & 0 & R_{1,2}^{2,1} & R_{1,2}^{2,2} & R_{1,2}^{2,3} \\ 0 & 0 & 0 & R_{1,3}^{2,1} & R_{1,3}^{2,2} & R_{1,3}^{2,3} \\ R_{2,1}^{1,1} & R_{2,1}^{1,2} & R_{2,1}^{1,3} & 0 & 0 & 0 \\ R_{2,2}^{1,1} & R_{2,2}^{1,2} & R_{2,2}^{1,3} & 0 & 0 & 0 \\ R_{2,3}^{1,1} & R_{2,3}^{1,2} & R_{2,3}^{1,3} & 0 & 0 & 0 \end{matrix}\right)$$

If we let$\text{R}_{3}^{\text{M}}=\left(\begin{matrix} R_{1,1}^{2,1} & R_{1,1}^{2,2} & R_{1,1}^{2,3} \\ R_{1,2}^{2,1} & R_{1,2}^{2,2} & R_{1,2}^{2,3} \\ R_{1,3}^{2,1} & R_{1,3}^{2,2} & R_{1,3}^{2,3} \end{matrix}\right)$, $\text{R}_{3}^{\text{F}}=\left(\begin{matrix} R_{2,1}^{1,1} & R_{2,1}^{1,2} & R_{2,1}^{1,3} \\ R_{2,2}^{1,1} & R_{2,2}^{1,2} & R_{2,2}^{1,3} \\ R_{2,3}^{1,1} & R_{2,3}^{1,2} & R_{2,3}^{1,3} \end{matrix}\right)$ and

$\text{K}_{3}\text{ }=\text{R}_{3}^{\text{M}}\cdot\text{R}_{3}^{\text{F}}=\left(\begin{matrix} \sum_{a=1}^{3} R_{1,1}^{2,a}R_{2,a}^{1,1} & \sum_{a=1}^{3} R_{1,1}^{2,a}R_{2,a}^{1,2} & \sum_{a=1}^{3} R_{1,1}^{2,a}R_{2,a}^{1,3} \\ \sum_{a=1}^{3} R_{1,2}^{2,a}R_{2,a}^{1,1} & \sum_{a=1}^{3} R_{1,2}^{2,a}R_{2,a}^{1,2} & \sum_{a=1}^{3} R_{1,2}^{2,a}R_{2,a}^{1,3} \\ \sum_{a=1}^{3} R_{1,3}^{2,a}R_{2,a}^{1,1} & \sum_{a=1}^{3} R_{1,3}^{2,a}R_{2,a}^{1,2} & \sum_{a=1}^{3} R_{1,3}^{2,a}R_{2,a}^{1,3} \end{matrix}\right)$,

Also here$R_{0}$was calculated as the dominant eigenvalue of the next generation matrix$\text{R}_{6}$. **R_6_** is a diagonal matrix with sub-matrices $\text{R}_{3}^{\text{M}}$and$\text{R}_{3}^{\text{F}}$. It can be shown that the determinant of **R_6_** is equal to the determinant of product of the sub-matrices, **K_3_** and that the dominant eigenvalue of **R_6_** is equal to the square root of the dominant eigenvalue of **K_3_.** The characteristic polynomial of a 3 by 3 matrix **K_3_** can be given by

$\det(K_{3})-c_{2}\lambda+trace(K_{3})\lambda^{2}-\lambda^{3}$ (9)

where det(**K_3_**) is the determinant of matrix **K_3_,** *c_2_* is the sum of the principal minors of the matrix and trace(**K_3_**) is equal to the sum of the diagonal elements of matrix **K_3_**. It was observed that the rows and columns of matrix **K_3_** are not linearly independent, implying that both det(**K_3_**) and *c_2_* are equal to zero. Thus Equation 9 reduces to$trace(K_{3})\lambda^{2}-\lambda^{3}$. Equating this equation to zero and solving for$\lambda$, we have $\lambda=0$ and $\lambda=trace(K_{3})$ as the solutions. Thus$R_{0}$, being the dominant eigenvalue of matrix **R**_6_ is given by $\sqrt{trace(K_{3})}=\sqrt{\sum_{a=1}^{3} R_{1,1}^{2,a}R_{2,a}^{1,1}+\sum_{a=1}^{3} R_{1,2}^{2,a}R_{2,a}^{1,2}+\sum_{a=1}^{3} R_{1,3}^{2,a}R_{2,a}^{1,3}}.$

Substituting for the $R_{g,a}^{g',a}$gives

$R_{0}=D\sqrt{\beta_{1}\beta_{2}\left(\sum_{a=1}^{3} f_{1,a}c_{1,a}\right)\left(\sum_{a=1}^{3} f_{2,a}c_{2,a}\right)}$. (10)

Under the assumption of proportionate mixing and same transmission probabilities by gender, $R_{0}$ (denoted$R_{0\_5i}$) is $R_{0\_5i}=D\beta\sqrt{\left(\sum_{a=1}^{3} f_{1,a}c_{1,a}\right)\left(\sum_{a=1}^{3} f_{2,a}c_{2,a}\right)}$

and under the assumption of proportionate mixing and different transmission probabilities by gender, $R_{0\_5ii}=D\sqrt{\beta_{1}\beta_{2}\left(\sum_{a=1}^{3} f_{1,a}c_{1,a}\right)\left(\sum_{a=1}^{3} f_{2,a}c_{2,a}\right)}.$

Under 100% assortative mixing, $R_{g,a}^{g',a'}=0$ for all $a\neq a'$and$f_{g,a}=1$ for all $g,a.$Assuming the same transmission probability by gender,

$R_{0\_5i}=D\beta\sqrt{c_{1,1}c_{2,1}}\text{ }$, $D\beta\sqrt{c_{1,2}c_{2,2}}$and $D\beta\sqrt{c_{1,3}c_{2,3}}$for the low, medium and high activity classes respectively.

Assuming different transmission probabilities by gender,

$R_{0\_5ii}=D\sqrt{\beta_{1}\beta_{2}c_{1,1}c_{2,1}}\text{ }$, $D\sqrt{\beta_{1}\beta_{2}c_{1,2}c_{2,2}}$ and$D\sqrt{\beta_{1}\beta_{2}c_{1,3}c_{2,3}}$ for the low, medium and high activity classes respectively.

### A method with different transmission probabilities by gender, fixed for the infectiousness period, allowing only heterosexual partnerships and one sexual activity class

This section addressed a method that incorporates different transmission probability by gender but ignores mixing i.e. it was based on one sexual activity class such that $c_{g,a}=c_{g}$ and that only heterosexual partnerships are formed. From the methods described in Section 4, the assumptions made in this section imply that $f_{g,a}=f_{g}=1$ for both $g=1,2.$ Substituting for the$f_{g,a}$and$c_{g,a}$ in Equation 8 gives

$$R_{0\_6}=D\sqrt{\beta_{1}\beta_{2}c_{1}c_{2}}.$$

**References**
